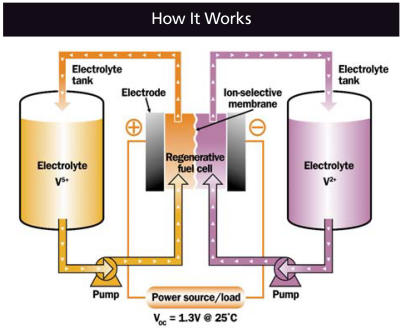# The Vanadium Advantage: Flow Batteries Put Wind Energy in the Bank

**DOI:** 10.1289/ehp.115-a358

**Published:** 2007-07

**Authors:** David C. Holzman

In absolute terms, wind is the second fastest growing energy source in the United States, behind natural gas. Worldwide, it is adding new capacity more than six times as fast as nuclear power, and grew by the equivalent of about 104 natural gas–fired plants (enough to serve 5.2 million U.S. homes)—in 2005 and 2006, according to the Worldwatch Institute. As of December 2006, total worldwide wind capacity stood at about 74 gigawatts, according to the Global Wind Energy Council in Brussels.

Wind power is touted for its lack of significant greenhouse gas emissions, water and air pollution, or radioactivity; anything that can increase wind power’s contribution toward meeting electricity needs will only further attenuate emissions of greenhouse gases and other pollutants.

Some experts are now looking to vanadium redox-flow batteries (VRBs) to provide the boost that wind power needs if it is to reach the next tier of capacity. Already these units are modulating wind power in several significant installations around the world. Most recently, VRB Power Systems of Vancouver, British Columbia, which has patented the technology, announced in May 2007 that the Australian government is investing AUS$1.83 million toward deploying VRBs in remote communities across Australia.

## Harnessing the Wind

The conventional wisdom about renewable sources of electricity—that storage is needed because of the intermittent nature of the resource—is true for solar, but much more so for wind. Wind power can be managed without storage if its contribution to the grid is small. “The grid itself acts as the most cost-competitive form of storage, with system managers turning up or down natural gas plants or the water flowing through a dam, depending on how strong the wind is blowing,” says Christine Real de Azua, assistant director of communications for the American Wind Energy Association.

But when wind becomes a player, at somewhere around 10% of a grid’s capacity, fluctuations in windspeed—which translate into fluctuations in the amount of electricity going into the grid—can become unmanageable. “Wind farms [without storage] are like a power station out of control,” says John Ward, director of Sorne Wind Energy and Tapbury Management in County Donegal, Ireland. The frequency of the current becomes unstable, major users start to be disconnected, and the network shuts down—just when the potential harvest is greatest.

This is why several states and Canadian provinces have banned expansion of wind power in their grids. This is also why Denmark, which produces wind power equivalent to about 20% of its electricity capacity, can use only about one-third of that electricity. Denmark must export the overflow to Norway, Sweden, and Germany, countries that can absorb Denmark’s excess while stanching their ample flows of hydropower when the Danish winds blow hard.

For other countries, though—particularly Britain and Ireland, with their island grids—sending electricity to other nations for storage and use is not an option. Enter the VRB, a battery first created at the University of New South Wales in the early 1980s. Proponents say VRBs are capable of storing large quantities of electricity at reasonable cost.

At King Island, off the coast of Australia, VRBs capable of furnishing 0.2 megawatt over four hours have boosted wind power’s contribution to that small, isolated grid from about 12% to 40%. The King Island VRB has also reduced the island’s net diesel emissions by an estimated 46% since 2004, in turn reducing CO_2_ emissions by more than 2,000 tons annually. In Sapporo, Japan, VRBs capable of generating 4 megawatts (with a pulse in demand up to 6 megawatts) for one and a half hours have been operating since January 2005. I n Castle Valley, Utah, the utility PacifiCorp has used VRBs for the last three years to maintain voltage on a 124-mile line servicing remote areas (analogous to maintaining fluid pressure in a pipeline) and to provide peak power. The PacifiCorp batteries can generate 0.25 megawatt over eight hours. By comparison, a typical natural gas–fired electric power plant can deliver about 250 megawatts, sufficient to supply about 50,000 average American homes.

In Ireland, Sorne Wind Energy, which has been running a 32-megawatt wind farm since July 2006, will install 12 megawatt-hours of VRB storage this year. A feasibility study jointly funded by Tapbury Management and Sustainable Energy Ireland estimated that at least 700 megawatts of storage would be required to boost Ireland’s wind base from the current 800 megawatts to the 3,000 megawatts that is currently contracted or proposed by the country’s major electric utility. Ward estimates that roughly one unit of storage is needed for every four units of capacity.

## Inside the VRB

Whereas a conventional battery stores chemical energy within an electrolyte solution, a VRB contains two different electrolyte solutions, each in a separate tank. In a charged VRB, one electrolyte is positively charged, and one is negatively charged. In order for the battery to provide power, the electrolytes flow through a fuel cell stack on opposite sides of a proton exchange membrane. Their opposite charges create a gradient that powers an external current.

Several characteristics unique to VRBs enable them to sustain utility-scale storage and power at potentially competitive prices. First, unlike conventional batteries, power output is independent from energy storage capacity—output depends on the size of the fuel cell stack, while the energy storage capacity depends on the size of the electrolyte tanks. Neither constrains the other, although the ratio of storage to power determines how long the batteries can run without recharging. Power can flow undiminished as long as there is fresh electrolyte to circulate through the stack.

VRBs, unlike many of their conventional counterparts, can be fully discharged without reducing life expectancy. In contrast, discharging a lead-acid battery more than 20–30% erodes longevity. Even under the best circumstances, lead-acid batteries are good for little more than 1,000 charge–discharge cycles. But a VRB in Sapporo has undergone around 14,000 cycles, says Dennis Witmer, director of the Arctic Energy Technology Development Laboratory at the University of Alaska, Fairbanks. The limiting factors are the proton exchange membrane and the pumps, both of which can be replaced. Discharged electrolyte can be replenished by running a current through the battery.

VRBs are far greener than other batteries, as they lack potentially toxic metals as lead, cadmium, zinc, and nickel, which can contaminate the environment at all phases of the conventional battery life cycle. VRBs’ most toxic component is the sulfuric acid of the electrolyte, which is only one-third as acidic as that in a lead-acid battery. But unlike lead-acid batteries, the electrolytes in a VRB function indefinitely, eliminating the disposal problem.

Vanadium itself has very low toxicity, and the batteries are designed to contain electrolyte spills. “We have the best environmental footprint of any storage technology,” says Simon Clarke, executive vice president for corporate development at VRB Power Systems.

For now, VRBs are not a viable option for cars. The energy density of gasoline equals 13,000 watt-hours per kilogram, while the typical VRB is still not much better than a lead-acid battery—about 40 watt-hours per kilogram, says Dan Lewis, research director of the Economic Research Council in London. Lithium ion batteries, as used in the latest generation of hybrid vehicles, have an energy density of about 200 watt-hours per kilogram.

Other types of flow batteries under development, such as those using vanadium bromide, could double the density of storage, but probably would still be inadequate for cars. Developing a sufficiently energy-dense flow battery would solve the problem of how long it takes to recharge currently available batteries for electric vehicles. Hypothetical automotive flow batteries could be replenished in minutes by replacing discharged electrolyte with freshly charged electrolyte.

As a plus, VRBs are not resource limited. The USGS’s estimate of the world vanadium resource is far greater than would be necessary to supply storage for total world electricity production.

## Cost Considerations

It’s hard to sort out the cost of electricity stored by flow batteries, because “there are too many variables,” says Robert B. Schainker, a technical executive with the Electric Power Research Institute, the research arm of the utility industry. Nonetheless, indications are favorable.

Ward expects wind power plus flow batteries to be plenty competitive in Ireland. Currently, he says, the production cost of natural gas–fired electricity there is 86 euros per megawatt-hour, or about 106 euros with the external cost of carbon emissions added in. For electricity from wind and VRBs, the floor price is 77 euros (equivalent to a little over 10 cents per kilowatt-hour, a not uncompetitive price in the United States). Nuclear is not slated to come to Ireland, but in Europe, production cost is about 100 euros per megawatt-hour.

Witmer calculates that providing electricity using VRBs, assuming 15,000 charge–discharge cycles, should cost 10 U.S. cents per kilowatt-hour—the more cycles, though, the lower the cost per kilowatt-hour. That would definitely prove competitive in off-grid Alaskan villages, where the rising cost of oil has pushed the fuel cost of diesel-fired electricity to around 16 to 17 cents per kilowatt-hour, he says.

The vagaries of the market for electricity can create additional profits for stored electricity, says Lewis. The value of electricity cycles daily with demand, from highs during peak use in the late afternoon and early evening, to lows during nocturnal troughs, meaning that electricity stored at night can be sold at a profit in the daytime. This can become even more advantageous in the winter in northwestern Europe, when demand usually peaks. Shortages can exaggerate the cycles, and have been known to boost spot market prices by an order of magnitude, according to Lewis.

At current prices, wind could contribute more than 30% of all electrical consumption in Ireland, says Ward, the limiting factor being the need to allow conventional power plants to live out their natural lives. In remote locations where electricity is diesel-fired, and in the long term, wind and VRBs could provide the majority of all electrical consumption, he says.

## Reaching Windspeed

VRB Power Systems, which purchased the rights to commercialize the batteries in much of the world, has built a dozen pre-commercial battery systems and placed them with key customers for evaluation. The company will begin shipping commercial projects midyear, says Clarke. Besides wind farms, potential markets include off-grid housing complexes and solar power plants, especially in places like California, where there is a large difference in the cost of peak and off-peak power. In addition, the company is focused on remote off-grid power supplies that are currently diesel-based, where the ability to cut diesel consumption makes storage financially appealing.

With growing interest in renewable energy worldwide, Ward says VRBs are poised to lift the lid on wind in the grid. Given concerns about climate change and declining availability of fossil fuels, this could be happening just in time.

## Figures and Tables

**Figure:**
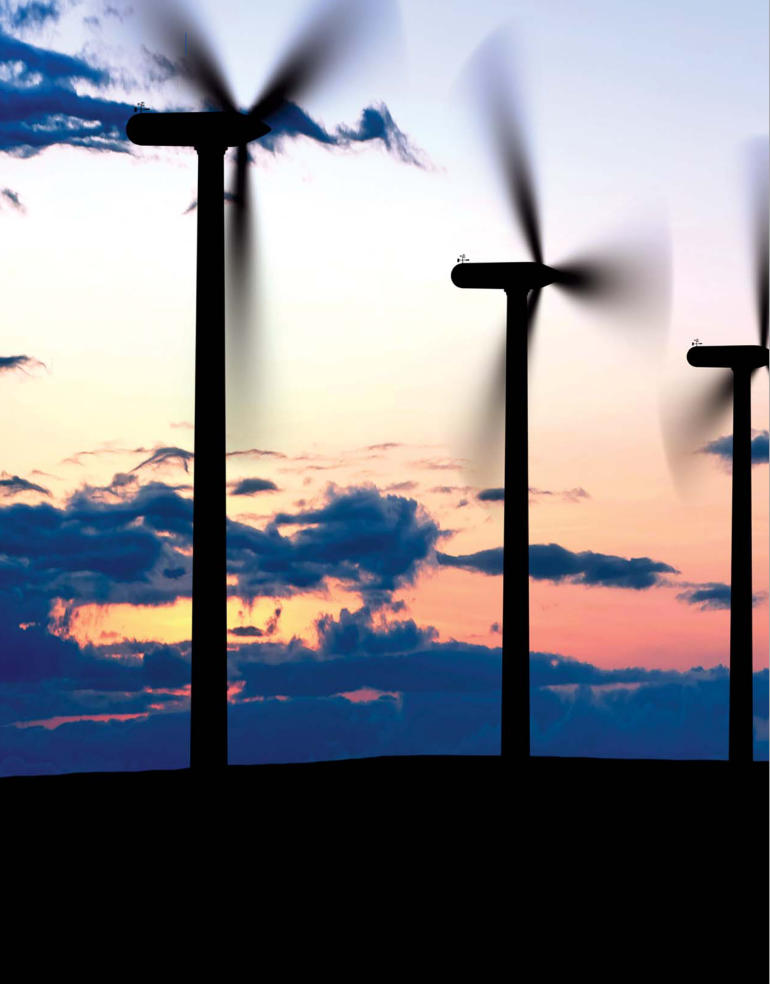


**Figure:**
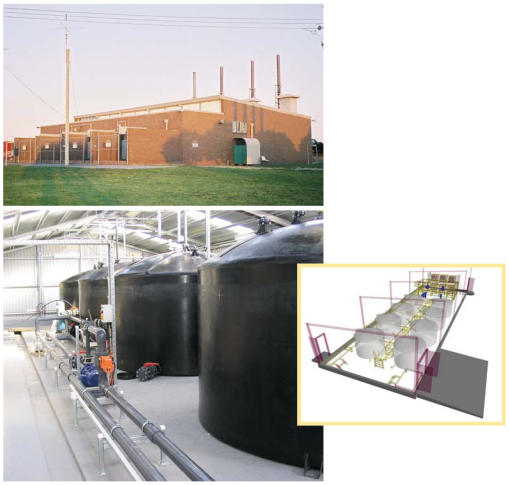
Tiger in the tank The unassuming exterior of the King Island VRB facility houses a bank of batteries that more than triple the island's wind capacity.

**Figure:**